# Linking Process and Outcome Measures to Improve Employment Support Programs for Individuals With the Most Significant Disabilities

**DOI:** 10.3389/fresc.2022.873568

**Published:** 2022-05-11

**Authors:** Tim Riesen, Corban Remund, Aubrey Snyder

**Affiliations:** Institute for Disability Research, Policy and Practice, Utah State University, Logan, UT, United States

**Keywords:** competitive integrated employment, most significant disabilities, process measures, outcome measures, accountability

## Abstract

Employment agencies and funding systems commonly use distal outcome measures such as employed or not employed, full-time or part-time, and continuous measures such as wage, hours worked, and type of job to document the employment status of individuals with disabilities. These measures continue to demonstrate that individuals with disabilities fall behind individuals without disabilities in all employment outcomes. While there is utility in distal outcome measures, it is difficult to determine what intervention or program variables were responsible for a specific outcome. Moreover, outcome measures do not provide sufficient information about the quality of employment supports and services an individual with disabilities receives. One way to improve accountability in employment support programs is to link outcomes to specific processes for obtaining and maintaining employment. The purpose of this manuscript is to describe how employment programs can link short-term (proximal) and long term (distal) outcomes measures to specific processes for employment. A customized employment framework is used to illustrate how systematically linking outcomes and processes improves accountability in programs that support job seekers with most significant disabilities.

## Introduction

The health of economies is often measured by the number of individuals who are attached to the workforce and who are actively employed. An examination of current economic outcome measures in the United States suggest that the U.S. is currently in a changing and robust labor market with unemployment hovering around 3.5% at the end of 2021 (1). Unfortunately, employment outcome measures indicate that the economic and employment realities for individuals with disabilities is quite different. The unemployment rate for individuals with disabilities is two times that of individuals without disabilities (7.9%) (1) while the American Community Survey (ASC) reports that only 37.8% of non-institutionalized individuals with disabilities between the ages of 21–64 are employed (2). When outcome data are drilled down by disability, individuals with the most significant disabilities (MSD) are even less likely to be employed. In fact, only 21.1% of individuals receiving day supports from state intellectual and developmental disability agencies are employed in competitive integrated employment (3).

These data are discouraging because research consistently demonstrates that when individuals with disabilities are engaged in competitive integrated employment (CIE), they experience improved outcomes in economic, psychological, and physical health factors (4). For example, individuals who participate in CIE earn more money (5, 6), tend to work more hours than their counterparts in sheltered work or group integrated work settings (7), and have more personal independence and self-determination (8, 9). Given what we know about the benefits of CIE, there has been a U.S. federal priority to increase high-quality job and career opportunities for individuals with significant barriers to employment, which includes individuals with MSD [e.g., (10–12)]. Despite these amendments, rules, and corresponding funding provisions to support individuals with MSD to find and maintain CIE, changes in employment outcomes remain slow and, in some cases, stagnate. Agencies and programs typically use distal employment outcome measures (i.e., employed or not employed, full-time or part-time, and continuous measures such as wage, hours worked, and type of job) to determine the success of employment programs and supports for individuals with disabilities. Unfortunately, relying exclusively on employment outcome measures does not provide sufficient information about what variables positively or negatively effect valued employment outcomes. Researchers, policy makers, and practitioners must adopt robust measurement strategies to ensure employment programs who support individuals with MSD formally link the taxonomy of an intervention or program to short-term (proximal) outcomes and long term (distal) outcomes. Ongoing adjustments to employment programs or interventions should be made based on evaluation of the process which in turn ensure practitioners are using validated strategies that help job seekers with MSD obtain and maintain meaningful work. The purpose of this article is to describe how employment programs can link proximal and distal outcomes to specific processes for employment. A customized employment framework will be used to illustrate how systematically linking outcomes and processes improves accountability in programs that support job seekers with MSD.

## Description of Current Outcome Measures

Both proximal and distal outcome measures are used to determine the impact of a program or intervention. Both measures attempt to answer, “what happened” after an intervention or program is implemented. Proximal outcome measures include data that is collected during program implementation and provide information about the most immediate and observable outcome of a program or intervention. Distal outcome measures include data that is collected after program or intervention implemented and are designed to determine the outcomes the program or intervention was intended to achieve (13). Policy makers, researchers, agencies commonly use distal outcome measures such as employed or not employed, type of job, benefited or not benefitted, hourly wage, monthly income, and hours worked per week to determine the success of an employment program.

The American Community Survey (ACS) is an example of a distal outcome measure that is used to inform policy. The ACS is an annual supplement to the U.S. Census and provides large-scale, aggregate distal outcome measures. The ACS provides information about demographics and social and economic statistics that serve as a base for the administration and evaluation of U.S. government programs (14). For disability related demographics, the ACS compiles data on six disability areas related to functional limitations in hearing, vision, cognition, ambulation, self-care, and independent living. For employment measures, the ACS examines employment status, number of weeks worked, and number of hours worked per week. According to Erickson (14), an individual is considered employed if one of two conditions are met. First, the individual works as a paid employee, works in his or her own business, works on his or her farm, or works 15 or more hours as an unpaid worker on a family farm or business. Second, the individual has a job but is not at work during the reference period (i.e., the individual was not working because of illness, bad weather, vacation, or other personal reasons). The ACS defines the reference period as the week prior to the ACS questionnaire being completed. The ACS also measures full-time/full-year employment. Full-time employment is defined as working 50–52 weeks in the previous 12 months and at least 35-h per week.

The Rehabilitation Services Administration (RSA) 911 data is an example of an agency specific distal outcome measure. The RSA-911 data is mandated by the Rehabilitation Act as amended by the Workforce Innovation and Opportunity Act (10) and is used to describe the performance of the vocational rehabilitation (VR) and supported employment (SE) programs in the annual report to the U.S. Congress (15). State VR agencies are required to submit RSA-911 data on a quarterly reporting period. RSA-911data submitted by each state VR agency is aggregated employment outcome data based on VR service recipient outcomes. Among many items, states report on demographics, service interventions (i.e., supported employment, customized employment), the hourly wage at the time an individual exits the program, hours worked, employment status (i.e., employed, not employed, registered apprenticeship), benefits received, and primary occupation using the Standard Occupational Classification.

## Process Measures

Process measures attempt to answer and document “how something happened” and provide a robust assessment of how well or the fidelity to which practitioners implement a program or intervention (16). The early use of process measures can be traced to improving manufacturing during World War I to monitor the quality of the manufacturing process (17) and the measurement construct has been adopted and is commonly used in the medical field to measure quality in specific medical procedures and treatments (18). Process measures help practitioners obtain actionable information to understand (a) what was done, (b) whether the action was done well (to fidelity), and (c) whether that action was implemented in a timely fashion (17). Using process measures requires systematic analysis of each process for a program or intervention and should be developed using empirical information rather than anecdotal observations of a specific process. Process measures should have demonstrated reliability and validity before they can be used as measures to improve performance and they should be connected and applied to both proximal and distal outcomes (19). Process measures may include information about what services the individual received, the fidelity to which the provider implemented a specific intervention or service, and whether the intervention or service aligns with validated practices. These measures do not guarantee change in outcomes, but they allow programs and practitioners to determine how a program or intervention is directly impacting proximal and distal outcomes. Systematically gathering process data allows programs to make meaningful adjustments to individualized employment programs and interventions that will increase overall distal outcomes. Unfortunately, the use of process measures in human services and rehabilitation fields is limited.

## Actionable Recommendation: Application for Job Seekers With MSD

For most working-age adults, the pathway to meaningful employment occurs over the lifespan (20), is based on a congruence between personality types and work environments (21), and is based on mutual interaction between the individual and the work environment (22). Finding and maintaining meaningful work is based on experiences and unfortunately, individuals with disabilities, especially those with MSD are more isolated and segregated (3, 23, 24) and are engaged in activities and supports that do not represent the demands of integrated community environments (25). As a result, individuals with MSD do not always engage in the full range of experiences that we know help build career identities and pathways and they need individually tailored interventions and supports to navigate, find, and maintain CIE (26). When employment programs measure distal outcomes only, they cannot be sure if changes in practices occurred and that job seekers with MSD are engaged in validated activities lead to CIE. Therefore, employment programs can ensure consistent and validated employment supports are implemented by adapting Donabedian's (27) approach to quality evaluation. Donabedian suggested that improvements in outcomes are made when a combination of measures including structure, process, and outcome measures are used to measure the quality of care. Using both process and outcome measures is important because they help connect a program or intervention to a specific outcome. Employment support programs can ensure program success by measuring and formally linking employment process measures to proximal and distal employment outcome and related measures.

### A Customized Employment Framework for Linking Process and Outcome Measures

Customized Employment (CE) represents a departure from traditional employment support methods and is designed to support individuals with MSD to find and maintain competitive integrated employment. CE is a sequential, cumulative process consisting of discovery, customized job development, and ongoing training and support. CE begins with discovery, which is psychosocial rehabilitation process used to determine an individual's strengths, interests, skills, and support needs to obtain and maintain customized employment (28). The discovery process includes interviews, observations, documentation review, and interactions with the job seeker (29). Discovery also uses observations of the employment seeker engaged in familiar and less familiar activities and requires interviews with family members and other influential persons in the job seeker's life. This information is used to develop well-coordinated customized job development activities. Customized job development activities use an informational interview framework to learn more about employers, working conditions, and other potential employers who engage in similar work. Jobs are then negotiated based on an employment proposal that accounts for the job seeker's unique skills and interest and the qualified employment specialist creates a job site analysis and plan.

Effective implementation of CE requires the qualified employment specialist to understand each component process of the discovery and customized job development. While it appears that qualified employment specialists are trained to implement critical components of CE, they are not implementing the components to fidelity (30). Integrating process measures with proximal and distal outcome measures can be used to ensure fidelity to intervention, evaluation, and adjusting components of the CE process. One way to measure the CE process is by using validated fidelity scales that have operationalized descriptions of what constitutes high-quality implementation for each element of the CE process. The Discovery Fidelity Scale (DFS) (31) and Job Development Fidelity Scales *(JDFS)* (32) are designed to operationalize the process for CE. The DFS was designed to measure fidelity to CE discovery best practices at both the systems and services levels. The systems fidelity measure examines processes for authorization and access, financing, and qualification of providers while the services fidelity measure examines the alignment of CE best practice to service implementation such as home and community observations, discovery activities, informational interviews, vocational profiles, and plans. The DFS has undergone several validation studies. First, Riesen et al. (33) used a three round, modified Delphi process to generate consensus about what experts believe are acceptable and not acceptable tenets of the DFS. The Delphi panel reviewed and rated the fidelity descriptors for discovery systems and services. The information obtained from the Delphi study was used to further refine the scale and Riesen et al. (28) conducted a study to determine the internal consistency of items on the DFS and the respective constructs. Results suggest that both the systems and services constructs have acceptable internal consistency. The final DFS consists of two subsections: discovery systems fidelity and discovery services fidelity. The systems section consists of five discovery system tenets and corresponding scaled fidelity descriptors. The services section consists of ten discovery services tenets and corresponding scaled fidelity descriptors. The scaled fidelity descriptors for each of the systems and services tenets represent levels of fidelity to the discovery process for each respective tenet.

The JDFS was designed to measure how to engage businesses that align with the job seeker's strengths and vocational interests. The JDFS consists of two sections: job development systems fidelity and job development services fidelity. Systems fidelity tenets cover the foundation for customized job development referrals; the incorporation of information gathered during discovery in the job development plans; and elements related to customized job development personnel, provider responsibilities, and transportation. Services fidelity tenets include building job-development plans based on discovery findings; using an informational interview approach to contact businesses; analyzing workplace cultures to ensure ecological fit; negotiating mutually beneficial and customized employment opportunities, including job creation through resource ownership or self-employment as appropriate; and maximizing opportunities for long-term career development and growth. Riesen et al. (34) used the Delphi method to build consensus among CE professionals about items on the JDFS and found that customized employment experts believed the fidelity descriptors had value when measuring fidelity to customized job development practice.

**Figure 1:**
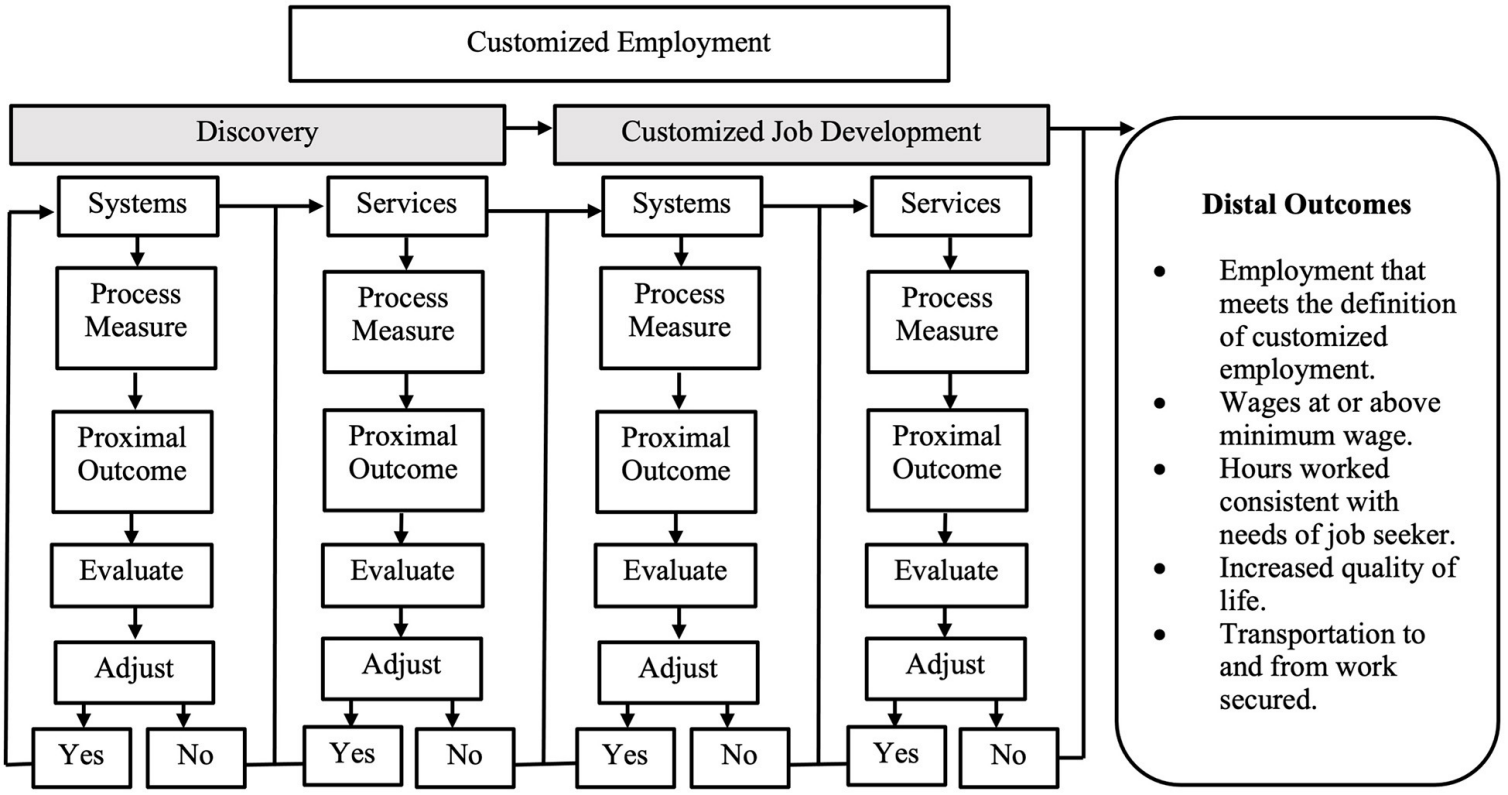
A conceptualization for integrating and linking processes and corresponding proximal and distal outcomes for CE.

A conceptualization for integrating and linking processes and corresponding proximal and distal outcomes for CE discovery and job development are outlined in Figure 1 and specific descriptions for the processes, process measures, and proximal and distal outcomes are in Table 1. As illustrated in the figure, discovery is the initial CE process with two components: systems and services. The systems processes ensure that discovery is appropriately authorized and financed while the services process ensures that discovery is appropriately implemented by a qualified employment specialist, the critical components of the discovery are followed, and the process accurately determines an individual's strengths, interests, skills, and support needs to obtain and maintain customized employment. For example, a critical discovery services process is to conduct home and neighborhood observations and observe the job seeker engaged in task-based activities. From the information gathered during these observations, the qualified employment specialist and the job seeker identify emerging vocational themes (i.e., career identity). Once themes are identified, the qualified employment specialist develops a list of potential employers that align with the identified vocational themes. Informational interviews are subsequently conducted with several employers to confirm if the type of work performed at the workplace aligns with the job seeker's interest and needs. The proximal outcome for this process is a fully developed vocational profile with an operational plan for customized job development. Linking process measures to proximal outcomes for both systems and service level discovery provides the necessary information to evaluate each process to determine whether specific adjustments to the process need to be made. If the process did not produce the desired proximal outcomes, adjustments are made to the process until the desired proximal outcome is achieved. If adjustments are not needed, the next service level process is implemented, evaluated, and adjusted.

**Table 1:** Process, process measures, and proximal and distal outcomes for CE discovery.

**Program**	**Process (31)**	**Process measures**
Customized Employment Discovery *(Systems)*	• Discovery is an alternative to vocational assessments and evaluations for persons eligible for CE and SE. • Discovery is part of CE or SE. • Discovery is accurately financed. • Providers give all eligible job seekers access to discovery, SE and CE. • The employment specialist provides all integrated employment phases.	• Number of authorizations for discovery for CE and SE eligible individuals. • Number of qualified agencies providing discovery. • Amount of funding allocated to discovery. • Percentage of job seekers engaged in discovery. • Percentage of employment specialists engaged in all employment phase. • Time engaged in discovery.
Customized Employment Discovery (*Services)*	• Begins discovery with an interview in the job seekers home or a mutually acceptable place in the community. • Observe and learn about the job seeker's personal spaces during interviews and visits to the job seeker's home • The employment specialist becomes familiar with the job seeker's neighborhood and surrounding area. • The employment specialist along with others observes the job seeker completing familiar activities, assisting if necessary. • The employment specialist and/or others assist the job seeker to complete several activities in unfamiliar places based on a determination of his or her vocational preferences and emerging vocational themes. • The employment specialist and the job seeker, to the extent possible, completes informational interviews with several businesses that align with the job seeker's, skills, tasks, interests, and vocational themes. • Review information collected to date, revisiting and/or including additional discovery information as needed, to develop the vocational profile. • A vocational profile narrative that completely describes the job seeker's discovery process, one that accurately reflects the job seeker, and answers: Who is this person? • The Employment specialist and the job seeker, along with other members of the job seeker's team, hold a discovery planning meeting to create an employment plan that contains businesses to contact for job development.	• Number of interviews conducted. • Summary of conversations. • Number and types of home observations. • Number and types of neighborhood observations. • Number and types of activity observations. • Number of activities related to employment. • Number and types of informational interviews. • Time to complete reviews. • Vocational profile is completed.
**Proximal outcomes**		
Increased number of consumers engaged in CE job development.		
Increased number of agencies who provide discovery.		
Completion of task-based discovery activities in the home or community location.		
Documented narrative descriptions of emerging patterns in employment.		
Completion of informational interviews with businesses that align with emerging vocational themes.		
Fully developed vocational profile outlining strengths, interests, and needs relate to employment.		
Fully develop plan for customized job development with information about potential employers.		
Schedule and hold an employment planning meeting to review vocational profile and job development plan to employment team.		
Benefits plan created.		
Accommodations and supports identified.		

After the discovery process is completed, a job seeker begins the process of customized job development. There are two components involved in the customized job development process: systems and services job development. Table 2 outlines the measures for customized job development. The job development systems process components ensure that customized job development is accurately funded, community partners necessary for successful employment are identified, and the jobseeker has access to adequate transportation at the same rate as other services provided. The services process and measures ensure that customized job development activities align with the job seeker's ideal conditions of employment and career identity. They also ensure that the employment specialist and job seeker are engaging meaningfully with businesses and documenting the types of tasks and activities performed at the business. The proximal outcomes for customized job development are a completed worksite and job task analysis, developed proposal for a customized job, and a fully developed job site training plan. Finally, distal outcome data is collected and examined to determine the overall efficacy of the CE process.

**Table 2:** Process, process measures, and proximal and distal outcomes for CE job development.

**Program**	**Process (32)**	**Process measures**
Customized Employment Job Development *(Systems)*	• Customized job development is based on the vocational profile. • Customized job development is based on dedicated financing to provide different services than traditional job development services. • The qualified employment specialist or a qualified person provides Customized job development. • The agency delivering Customized job development services ensures relationships, based on the discovery plan, between the job seeker and community members • The job seeker, utilizing customized job development services, has accessible and reliable transportation services and financing	• Number of qualified agencies providing customized job development. • Amount of funding allocated to customized job development. • Number of community partnerships. • Type of job seeker information presented to employer. • Type of financial planning available to job seeker. • Number of meetings with business that align with strengths, interest, and needs. • Time engaged in job development. • Types of worksite analysis.
Customized Employment Job Development (*Services)*	• The qualified employment specialist and the job seeker decide which of the positive skills, assets, supports, information, pictures or videos, learned or developed during discovery, will be shared with employers and, if applicable, used to develop small business ownership. • The qualified employment specialist assists the job seeker to develop employment that meets the expectations of the financial plan developed during discovery that includes goals and resources, information from a benefits planner, and if applicable, plans to ensure the financial success of job seeker's own business. • The qualified employment specialist and the job seeker work together developing employment that meets the ideal number of hours of work each week and the number of hours of non-work services and supports. • The qualified employment specialist and the job seeker meet with businesses to identify a fit between the workplace culture, tasks, skills, and the job seeker's ideal conditions for employment. • The qualified employment specialist, always with the job seeker when possible, conduct informational interviews with businesses. • The qualified employment specialist completes formal analyses of job tasks, skills, coworker supports, and employee training. • The qualified employment specialist negotiates a mutually beneficial economic win-win proposal, between the job seeker and the business, or when applicable, a self-employment proposal. • The qualified employment specialist creates a job site training plan, detailing job tasks, required skills, new skill development, training, and support strategies for the employer.	• Number of interviews conducted. • Summary of conversations. • Number and types of home observations. • Number and types of neighborhood observations. • Number and types of activity observations. • Number of activities related to employment. • Number and types of informational interviews. • Time to complete reviews. • Vocational profile is completed.
**Proximal outcomes**		
Fully funded customized job development activities.		
Adequate transportation commensurate with day service transportation.		
Community partners identified.		
Documentation that identifies job seekers ideal conditions of employment and business.		
Completed worksite and job task analysis.		
Completed proposal for a customized job at the job seekers ideal place of employment.		
Fully developed job site training plan.		
**Distal outcomes**		
Employment that meets the definition of customized employment.		
Improved Quality of Life measures.		
Employment that aligns with the job seeker's career identity.		
Wages at or above minimum wage.		
Hours worked consistent with needs of job seeker.		
Transportation to and from work secured.		
Long-term supports secured.		
Natural supports secured.		

## Discussion

The perennially low employment rates for individuals with MSD underscore the importance of expanding, connecting, and balancing measures used to determine the quality and success of employment programs for individuals with MSD. The most common framework for determining success of employment programs relies on distal outcome measures. Unfortunately, relying only on distal employment outcomes as an indicator of program success is problematic because distal outcomes are often influenced by other non-intervention/program factors (16, 35). Without objectively measuring fidelity to the process and linking the process to proximal and distal outcomes, we run the risk of agencies and practitioners believing they are implementing effective programs when they are not. Therefore, as researchers, policy makers, funding agencies, and practitioners examine how to improve outcomes for individuals with MSD, they should adopt more balanced measures to determine the efficacy of employment support programs and interventions. From a research and policy prospective, using both process and outcome measures ensures that employment support practices are operationalized and replicable. From a funding perspective, a balance set of process and outcome measures provides funding agencies a mechanism to continually evaluate the efficacy of the programs and invest in programs that demonstrate positive process and outcome measures for individuals with MSD. Finally, from a practitioner perspective, linking process and outcome measures allows practitioners to continually assess the program or intervention so that meaningful adjustments can be made.

## Author Contributions

CR and AS contributed to the manuscript by conceptualizing and writing specific sections. All authors contributed to the article and approved the submitted version.

## Conflict of Interest

The authors declare that the research was conducted in the absence of any commercial or financial relationships that could be construed as a potential conflict of interest.

## Publisher's Note

All claims expressed in this article are solely those of the authors and do not necessarily represent those of their affiliated organizations, or those of the publisher, the editors and the reviewers. Any product that may be evaluated in this article, or claim that may be made by its manufacturer, is not guaranteed or endorsed by the publisher.

## References

[1] US Bureau of Labor Statistics. News Release. (2022). Available online at: https://www.bls.gov/news.release/pdf/empsit.pdf

[2] Erickson W Lee C von Schrader S. Disability Statistics from the American Community Survey (ACS). Ithaca, NY: Cornell University Yang-Tan Institute (YTI) (2017). Available online at: www.disabilitystatistics.org

[3] Winsor J Timmons J Butterworth J Migliore A Domin D Zalewska A. StateData: The National Report on Employment Services and Outcomes Through 2018. Boston, MA: University of Massachusetts Boston, Institute for Community Inclusion (2021).

[4] Taylor J Avellone L Brooke V Wehman P Inge K Schall C. The impact of competitive integrated employment on economic, psychological, and physical health outcomes for individuals with intellectual and developmental disabilities. J Appl Res Intellect Disabil. (2022) 35:448–59. 10.1111/jar.12974 34994035

[5] Blick RN Litz KS Thornhill MG Goreczny AJ. Do inclusive work environments matter? Effects of community-integrated employment on quality of life for individuals with intellectual disabilities. Res Dev Disabil. (2016) 53–4:358–66. 10.1016/j.ridd.2016.02.015 26977937

[6] Butterworth J Hiersteiner D Engler J Bershadsky J Bradley V. National Core Indicators©: Data on the current state of employment of adults with IDD and suggestions for policy development. J Vocat Rehabil. (2015) 42:209–20. 10.3233/JVR-150741

[7] Cimera RE. Does being in sheltered workshops improve the employment outcomes of supported employees with intellectual disabilities? J Vocat Rehabil. (2011) 35:21–7. 10.3233/JVR-2011-550

[8] Schall C Sima AP Avellone L Wehman P McDonough J Brown A. The effect of business internships model and employment on enhancing the independence of young adults with significant impact from autism. Intellect Dev Disabil. (2020) 58:301–13. 10.1352/1934-9556-58.4.301 32750714

[9] Shogren K Weymeyer ML Palmer SB Rifenbark GG Little TD. Relationship between self-determination and post-school outcomes for youth with disabilities. J Spec Educ. (2015) 48:256–67. 10.1177/0022466913489733

[10] Workforce Innovation and Opportunity Act of 2014. No. 113–128 U.S. Congress. (2014). Available online at: https://www.govinfo.gov/app/details/PLAW-113publ128

[11] Individuals Individuals with Disabilities Education Improvement Act of 2004 20, U,.S.C. §1400. (2004). Available online at: https://sites.ed.gov/idea/statute-chapter-33/subchapter-i/1400

[12] Home and Community Based Servicers Final Settings Rule 79 FR §2947. Office of the Federal Register, National Archives and Records Administration (2014).

[13] Lewis CC Boyd MR Walsh-Bailey C Lyon AR Beidas R Mittman B. A systematic review of empirical studies examining mechanisms of implementation in health. Implement Sci. (2020) 15:1–25. 10.1186/s13012-020-00983-3 32299461 PMC7164241

[14] Erickson W. A Guide to Disability Statistics From the American community Survey (2008 forward). (2012). Available online at: https://ecommons.cornell.edu/bitstream/handle/1813/90090/DE141_PDF2.pdf?sequence=1andisAllowed=y

[15] US Department of Education. Office of Special and Rehabilitation Services. Policy Directive 19-03. (2019). Available online at: https://rsa.ed.gov/sites/default/files/subregulatory/pd-19-03.pdf

[16] Kessell E Pegany V Keolanui B Fulton BD Scheffler RM Shortell SM. Review of Medicare, Medicaid, and commercial quality of care measures: considerations for assessing accountable care organizations. J Health Polit Policy Law. (2015) 40:761–96. 10.1215/03616878-3150050 26124294

[17] Crombie IK Davies HTO. Beyond health outcomes: the advantages of measuring process. J Eval Clin Pract. (1997) 4:31–8. 10.1046/j.1365-2753.1998.t01-1-00003.x 9524910

[18] Leeds IL Ladd MR Sundel MH Fannon ML George JA Boss EF. Process measures facilitate maturation of pediatric enhanced recovery protocols. J Pediatr Surg. (2018) 53:2266–72. 10.1016/j.jpedsurg.2018.04.037 29801659 PMC8710141

[19] Watkins KE Paddock SM Hudson TJ Ounpraseuth S Schrader AM Hepner KA. Association between process measures and mortality in individuals with opioid use disorders. Drug Alcohol Depend. (2017) 177:307–14. 10.1016/j.drugalcdep.2017.03.033 28662975 PMC5557034

[20] Super DE. A life-spa approach to career development. J Vocat Behav. (1980) 16:282–98.12808355

[21] Holland JL. Exploring careers with typology: What we have learned and some new directions. Am Psychol. (1996) 51:397–406. 10.1037/0003-066X.51.4.397

[22] Dawis RV Lofquist LH. A psychological theory of work adjustment: An individual difference model and its application. Minneapolis, MN: University of Minnesota (1984).

[23] Bates-Harris C. Segregated and exploited: The failure of the disability service system to provide quality work. J Vocat Rehabil. (2012) 36:39–64. 10.3233/JVR-2012-0581

[24] Merrels J Buchanan A Waters R. “We feel left out”: experiences of social inclusion for the perspective of young adults with intellectual disabilities. J Intellect Dev Disabil. (2019) 44:13–22. 10.3109/13668250.2017.1310822

[25] Thibedeau Boyd JM Beckman CJ. Stop Making It Weird 2.0: Imagining a less-weird world. J Vocat Rehabil. (2019) 50:301–5. 10.3233/J.V.R.-191011

[26] Wehman P Taylor J Brooke V Avellone L Whittenburg H Ham W. Toward competitive employment for persons with intellectual and developmental disabilities: What progress have we made and where do we need to go. Res Pract Pers Sev Disabil. (2018) 43:131–44. 10.1177/1540796918777730

[27] Donabedian A. Evaluating the quality of medical care. Milbank Q. (2005) 83:691–729. 10.1111/j.1468-0009.2005.00397.x 16279964 PMC2690293

[28] Riesen T Hall S Keeton B Snyder A. Internal consistency of the customized employment discovery fidelity scale: a preliminary study. Rehabil Couns Bull. (2021) 43:183–93. 10.1177/00343552211043259

[29] WINTAC. The essential elements of customized employment for universal application. Workforce Innovation Technical Assistance Center. (2017). Available online at: https://leadcenter.org/wp-content/uploads/2017/07/The-Essential-Elements-of-Customized.pdf

[30] Inge K Sima AP Riesen T Wehman P Brookes-Lane N. A Gap Analysis Of Practitioner Knowledge Of Customized Employment Practices. Rehabilitation Counseling Bulletin (in press).

[31] Hall S Keeton B Cassidy P Iovannone R Griffin C. Discovery Fidelity Scale. Atlanta, GA: Center for Social Capital. Available online at: https://www.griffinhammis.com/wp-content/uploads/2020/10/DFS-December-2018-4-2.pdf

[32] Hall SR Keeton B. Job Development Fidelity Scale. Griffin-Hammis Associates (2019).

[33] Riesen T Hall S Keeton B Jones K. Customized employment discovery fidelity: Developing consensus among experts. J Vocat Rehabil. (2019) 50:23–37. 10.3233/JVR-180985

[34] Riesen T Hall S Keeton B Snyder A. Building consensus among experts regarding customized job development fidelity descriptors: a delphi study. J Rehabil. (2021) 87:22–30.

[35] Brenner MH Curbow B Legro MW. The proximal-distal continuum of multiple health outcome measures: the case of cataract surgery. Med. Care. (1995) 33:AS236–44. 7723452

